# How Do High-Performance Work Systems Affect Individual Outcomes: A Multilevel Perspective

**DOI:** 10.3389/fpsyg.2018.00586

**Published:** 2018-04-24

**Authors:** Junwei Zhang, M. Naseer Akhtar, P. Matthijs Bal, Yajun Zhang, Usman Talat

**Affiliations:** ^1^College of Economics and Management, Huazhong Agricultural University, Wuhan, China; ^2^Department of Management and Human Resource, NUST Business School, National University of Sciences and Technology, Islamabad, Pakistan; ^3^Lincoln International Business School, University of Lincoln, Lincoln, United Kingdom; ^4^School of Business Administration, Guizhou University of Finance and Economics, Guiyang, China; ^5^Salford Business School, University of Salford, City of Salford, United Kingdom

**Keywords:** high-performance work systems, organization-based self-esteem, line managers’ goal congruence, job performance, job satisfaction

## Abstract

Research on high-performance work systems (HPWS) has suggested that a potential disconnection may exist between organizational-level HPWS and employee experienced HPWS. However, few studies have identified factors that are implied within such a relationship. Using a sample of 397 employees, 84 line managers, and 21 HR executives in China, we examined whether line managers’ goal congruence can reduce the difference between organizational-level HPWS and employee experienced HPWS. Furthermore, this study also theorized and tested organization-based self-esteem (OBSE) as a mediator in the associations between employee experienced HPWS and job performance and job satisfaction. Using multilevel analyses, we found that line managers’ goal congruence strengthened the relationship between organizational-level HPWS and employee experienced HPWS, such that the relationship was significant and positive when line managers’ goal congruence was high, but a non-significant relationship when line managers’ goal congruence was low. Moreover, employee experienced HPWS indirectly affected job performance and job satisfaction through the mechanism of OBSE beyond social exchange perspective.

## Introduction

Research on strategic human resource management (SHRM) has suggested that high-performance work systems (HPWS) enable firms to become more effective and gain core competitive advantage (Bowen and Ostroff, 2004; Takeuchi et al., 2007; Liao et al., 2009). HPWS are defined as a group of internally coherent and consistent HR practices that are designed to promote employee competence, motivation, as well as commitment (Datta et al., 2005).

The content approach of HPWS posits that HPWS are associated with enhanced subjective and objective performance (Guthrie, 2001; Sun et al., 2007; Aryee et al., 2012) as HPWS encompass related HR practices that can improve employee knowledge, skills, and motivations (Piening et al., 2014; Sanders et al., 2014). Many empirical studies have also found that HPWS are linked to various desirable outcomes, such as better job performance, creativity, and innovation (Jiang et al., 2013; Chang et al., 2014; Costantini et al., 2017), more organizational citizenship behavior (Kehoe and Wright, 2013), greater organizational commitment and job satisfaction (Messersmith et al., 2011; Korff et al., 2017), higher organizational performance, and lower employee turnover rates (Huselid, 1995; Sun et al., 2007; Jiang et al., 2012). However, some researchers have also challenged the validity of these findings. For instance, Wright et al. (2005) reported that the positive effect of HPWS on firms’ future operational and financial performance disappears when controlling for past or concurrent performance. Combs et al. (2006) conducted a meta-analysis of 92 studies and discovered only a moderate relationship between HPWS and organizational performance (*r* = 0.20). To clarify these inconclusive arguments and findings, scholars have advocated the process perspective of HPWS. This view argues that excellent HR systems designed by organizations may not suffice to positively affect employee performance if such systems can’t be perceived, understood, and accepted by employees (Katou et al., 2014; Sanders et al., 2014). A crucial reason why employees lack accurate perceptions and understanding of HPWS is that their line managers fail to effectively implement HR practices. Therefore, it is imperative to explore whether HPWS that firms design are consistent with employee perceived HPWS and how to reduce this difference.

Although previous research has almost exclusively focused on organizational -level HPWS, an emerging stream of work has suggested that organizational-level HR practices may not be applied uniformly across employee groups (Liao et al., 2009; Aryee et al., 2012). That is, a possible disconnection can exist between organizational-level HPWS and employee experienced HPWS. Organizational-level HPWS refer to HR systems that firms develop and implement, not only on paper (Den Hartog et al., 2013), but also to manage employees and redesign work systems. Organizational-level HPWS reflect the goals and intentions of organizations as they involve the decisions about how organizations manage their employees (Den Hartog et al., 2013). This inconsistency will lead employees to inaccurately understand the goals of organizations and to engage in behaviors that deviate from the strategic intentions of organizations. Therefore, it is critical to explore the causes of this misalignment. However, few studies have identified factors that narrow the gap between organizational-level and employee experienced HPWS. As Nishii and Wright (2008) suggested, “work group leaders likely implement HR policies quite differently, yet we know little as to what might explain the result from such differences” (p. 239). Line managers undertake more HR responsibilities (e.g., recruitment, training, performance appraisal, and promotion) today than in the past (Jiang, 2013; Kuvaas et al., 2014). Line managers may acquire HPWS information from HR departments and implement and convey HR practices to employees. As a result, line managers play a vital role in implementing HPWS (Bos-Nehles et al., 2013; Brewster et al., 2013; Sikora et al., 2015; Pak and Kim, 2016). Research has also argued that line managers are increasingly recognized as the agents of organizations to enforce HR practices in their groups (e.g., Kulik and Bainbridge, 2006; Purcell and Hutchinson, 2007). Furthermore, Sanders et al. (2014) called for research to investigate the roles of line managers in transferring HR information from the top down. Thus, we propose the relationship between organizational-level HPWS and employee experienced HPWS may be contingent upon line managers’ characteristics (i.e., goal congruence with their organizations).

To better elucidate the impacts of employee experienced HPWS on individual outcomes, research has predominantly drawn from social exchange theory to explicate the underlying mechanism in the HPWS literature (Takeuchi et al., 2007; Messersmith et al., 2011; Kehoe and Collins, 2017). Social exchange theory suggests that when employees receive benefits from the organization, they are likely to reciprocate with their behaviors and attitudes valued by the organization (Blau, 1964). HPWS including HR practices such as training, developmental performance management, and compensation reflect organizations’ investment in employees (Liao et al., 2009). Consequently, when firms offer HPWS to employees, employees would reciprocate by demonstrating positive behaviors and attitudes such as job performance, job satisfaction, as well as organizational commitment (Messersmith et al., 2011; Kehoe and Wright, 2013; Korff et al., 2017). The focus of the social exchange perspective is that HPWS help to form long-term employee-employer exchange relationship. In addition to exchanging important resources, employment relationship may carry more meanings for employees because work is a medium through which employees can not only get economic and social resources, but also gain esteem and the sense of personal accomplishment (Liu et al., 2013b). Hence, we further argue that the effects of HPWS are not just about reciprocation (i.e., social exchange), but also crucially about influencing self-worth perceptions. However, this perspective has been neglected in the HPWS research. Organization-based self-esteem (OBSE) refers to the self-perceived value that individuals have of themselves as organization members acting within an organizational context (Pierce et al., 1989). We suggest that when HPWS are granted, employees would feel that organizations value them. This granted HPWS may foster employee OBSE. Thus, our study is rooted in self-concept-based theory (Shamir et al., 1993; Chan et al., 2013) to theorize how OBSE bridges the relationships between employee experienced HPWS and job performance and job satisfaction by establishing its incremental validity over the social exchange approach.

Overall, our study contributes to the HPWS literature by hypothesizing and testing how line managers’ goal congruence narrows the gap between organizational-level HPWS and employee experienced HPWS. Furthermore, drawing from self-concept-based theory, we theorize and examine OBSE as an additional explanatory mechanism in the relationships between employee experienced HPWS and job performance and job satisfaction beyond social exchange perspective.

## Theoretical Overview and Hypotheses Development

### Organizational-Level High-Performance Work Systems and Employee Experienced High-Performance Work Systems

In this study, we focus on HPWS including comprehen sive recruitment, rigorous selection, extensive training, developmental performance management, performance-based compensation, flexible job design, participative decision-making, and information sharing. All of these dimensions have been used in the prior HPWS literature (Sun et al., 2007; Jiang, 2013). Previous research has theorized and proposed the conceptualization of HPWS primarily based on the system view because HPWS can create mutually reinforcing, synergistic effects (Rabl et al., 2014). Thus, the rationale underlying the HPWS research is that the synergistic effects of HPWS are stronger than the sum of the effects of the individual ones (Subramony, 2009; Aryee et al., 2012). Hence, we analyze HPWS in line with dominant views postulating that HR practices should be regarded as synergetic.

At the start of SHRM research, HPWS were identified at the organizational level, assessed by general managers and HR managers (Sun et al., 2007; Chang et al., 2014). Now, some researchers pay attention to the employee perceived HPWS because HR practices of organizations are likely to have salutary effects on employees’ attitudes and behaviors only when they are perceived, understood, and accepted by employees (Kehoe and Wright, 2013; Piening et al., 2013; Boon and Kalshoven, 2014). However, research has suggested that the misalignment between organizational-level HPWS and employee experienced HPWS may occur. For instance, Liao et al. (2009) showed a non-significant relationship between manager-rated HPWS and employee perceived HPWS. Nishii and Wright (2008) proposed that designed HR practices would not be necessarily implemented in organizations, which results in inaccurate employee perceptions and poor understanding of HPWS. Aryee et al. (2012) and Den Hartog et al. (2013) indicated that branch-level HPWS positively relate to employee experienced HPWS. As a consequence, it is of great significance to examine the factors that enhance the alignment of organizational-level HPWS with employee experienced HPWS.

Social information processing theory provides a theoretical explanation for the relationship between organizational-level HPWS and employee experienced HPWS. This theory postulates that employees tend to use information collected from social environment to guide their perceptions, attitudes, and behaviors (Salancik and Pfeffer, 1978). Organizational-level HPWS offer the contextual cues for employees to shape their perceptions of HR practices. However, the argument that organizational-level HPWS relate to employee perceived HPWS implicitly assumes that line managers should effectively implement HPWS espoused by the organization and transmit HR information to employees. Line managers, as the immediate and the most important social context of employees, play a critical role in shaping employee experienced HPWS (Sikora et al., 2015; Pak and Kim, 2016). Thus, we propose line managers’ goal congruence as a moderator elucidating when organizational-level HPWS affect employee experienced HPWS. Line managers’ goal congruence refers to the extent to which line managers’ personal goals are consistent with organizational goals (Vancouver and Schmitt, 1991; Ozcelik, 2013).

### The Moderating Effect of Line Managers’ Goal Congruence

Previous studies have suggested that perceived goal congruence may have positive effects on individual attitudes and behaviors, such as job satisfaction, organizational commitment, job performance, and job engagement (Vancouver and Schmitt, 1991; Kristof-Brown and Stevens, 2001; Bouckenooghe et al., 2015).

The congruence of line manager-organization goal implies that there are no agency conflicts that may exist between line managers’ personal interests and those of their organizations. In other words, when line managers’ goals are in line with the organization’s goals, mutually beneficial outcomes are likely to occur (Ozcelik, 2013). In addition, the implementation of HPWS is an effective way to reach organizational goals (Becker and Gerhart, 1996). Consequently, when line managers’ personal goals are consistent with those of their organizations, they may be devoted to implementing HPWS (Kristof-Brown and Stevens, 2001; De Clercq et al., 2014). Furthermore, when employees have any questions about HPWS, line managers with high goal congruence may communicate with them more effectively. Hence, the discrepancy between organizational-level HPWS and employee experienced HPWS is likely to be diminished for employees working with line managers with high goal congruence because they are able to effectively implement HPWS and deliver HR information to employees.

Conversely, low level of goal congruence creates goal conflicts between line managers and their organizations. Hence, line managers with low goal congruence are likely to weigh priorities between personal interests and organizational goals. Existing research revealed that employees who experience goal conflicts with their supervisors may invest less effort to achieve organizational goals, and engage instead in behaviors that harm the organizations (De Clercq et al., 2014). In other words, line managers with low goal congruence may perfunctorily implement HPWS, and in turn accrue the disconnection between organizational-level HPWS and employee experienced HPWS. Based on above arguments, we hypothesize:

*Hypothesis 1:* Line managers’ goal congruence moderates the relationship between organizational-level HPWS and employee experienced HPWS, such that the relationship is stronger when line managers’ goal congruence is high than when line managers’ goal congruence is low.

### Employee Experienced High-Performance Work Systems, Organization-Based Self-Esteem, and Individual Outcomes

In addition to the extant research that focused on social exchange as a mediator of the relationship between HPWS and establishment performance (Takeuchi et al., 2007), we introduce employee OBSE as an explanatory mechanism in the relationships between employee experienced HPWS and job performance and job satisfaction. OBSE refers to the self-perceived value that individuals have of themselves as organization members acting within an organizational context (Pierce et al., 1989). Global self-esteem is not limited to a specific realm (Pierce and Gardner, 2004; Ceschi et al., 2017). OBSE is developed based on global self-esteem and represents individual self-esteem in the organizational context. Our study focuses on OBSE rather than global self-esteem because previous work has found that OBSE has stronger predictive power for organization-related construct (Pierce et al., 1989; Chan et al., 2013). Scholars have identified that OBSE is subject to some contextual and individual enablers, such as perceived organizational support, organizational justice, delegation, leader-member exchange, self-efficacy, and internal locus of control (McAllister and Bigley, 2002; Pierce and Gardner, 2004; Chen and Aryee, 2007; Lee and Peccei, 2007; Ferris et al., 2009; Liu et al., 2013a). Prior research has also found that OBSE is linked to several individual outcomes, such as enhanced job performance and organizational citizenship behavior, increased affective commitment and job satisfaction (Pierce and Gardner, 2004; Chen and Aryee, 2007; Bowling et al., 2010; Sun et al., 2014).

Self-concept-based theory helps to explain how employee experienced HPWS contribute to employee job performance and job satisfaction through the mediating role of OBSE (Shamir et al., 1993). This theory postulates that employees who interact with a significant other may internalize significant other’s evaluation for them to form their self-concept (Shamir et al., 1993; Chan et al., 2013). The organization as employees’ significant other, may greatly affect employee self-evaluation. More specifically, HPWS including HR practices such as training and development reflect organizational investment in employees (Takeuchi et al., 2007; Liao et al., 2009), which makes employees perceive that organizations consider them to be important and valued. Furthermore, HR practices such as performance-based compensation and developmental performance management demonstrate organizational recognition and consideration for employee contributions (Liao et al., 2009). Flexible job design enables employees to perceive that they can control their work and decide how to do and what to do, which represents organizational trust and appreciation for employees (Takeuchi et al., 2007). Participative decision-making shows organizational respect for employees’ suggestions (Liao et al., 2009). The incorporation of such positive messages (e.g., organizational recognition, trust, and respect) into the employee’s self-concept promotes employee OBSE (Chen and Aryee, 2007). Overall, we postulate that HPWS positively predict employee OBSE.

We further argue that OBSE will lead to better job performance and job satisfaction. The extant study has adopted self-concept-based theory to illuminate how OBSE affects its presumed outcomes (Shamir et al., 1993; Chan et al., 2013). This theory advocates that individuals are prone to maintain self-consistency, and motivating them to act in a manner that reinforces their self-concept (Shamir et al., 1993). Employees with high OBSE perceive that they are able, valued, and meaningful. According to self-concept-based theory, these employees are likely to exhibit positive behaviors and attitudes (e.g., job performance, job satisfaction) to strive for self-consistency. Many empirical studies have also revealed the positive relationships between OBSE and job performance and job satisfaction (Pierce et al., 1989; Chen and Aryee, 2007; Liu et al., 2013a). Thus, we predict:

*Hypothesis 2a:* OBSE mediates the relationship between employee experienced HPWS and employee job performance.
*Hypothesis 2b:* OBSE mediates the relationship between employee experienced HPWS and employee job satisfaction.

Combined with Hypothesis 1 that states the joint effect of organizational-level HPWS and line managers’ goal congruence on employee experienced HPWS, we propose a serial mediated moderation model in which employee experienced HPWS and OBSE sequentially mediate the impacts of the interaction between organizational-level HPWS and line managers’ goal congruence on employee job performance and job satisfaction. Thus, we posit the following:

*Hypothesis 3a:* The interaction between organizational-level HPWS and line managers’ goal congruence indirectly affects employee job performance sequentially through employee experienced HPWS and OBSE.
*Hypothesis 3b:* The interaction between organizational-level HPWS and line managers’ goal congruence indirectly affects employee job satisfaction sequentially through employee experienced HPWS and OBSE.

**FIGURE 1:**
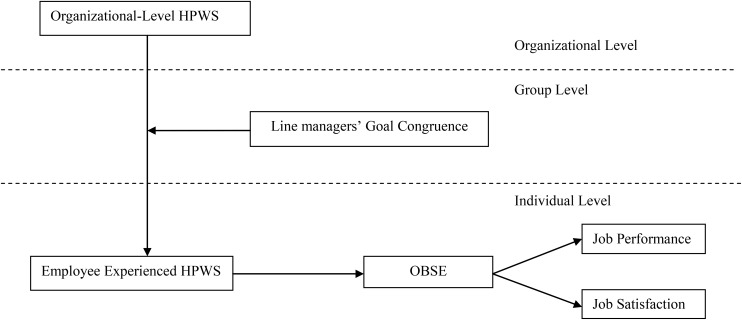
Theoretical model.

In summary, the research model is presented in **Figure 1**.

## Materials and Methods

### Sample and Procedures

Our data were collected from 21 companies located in the People’s Republic of China. The participating organizations belonged to the software development, manufacturing, and electric power generation industries. To control for common method bias, questionnaires were developed and administrated to employees, line managers, and HR executives. A cover letter attached to each questionnaire explained that participation was voluntary, and that the purposes of the survey were only for research, and that the confidentiality of their responses was assured. Employees completed a survey including items tapping employee experienced HPWS, OBSE, job satisfaction, and social exchange. Line managers provided the assessment of job performance for their subordinates and their goal congruence with organizations. Furthermore, HR executives rated HPWS that the firms implemented. Prior to collecting the study data, we contacted HR executives in each company and asked them to randomly pick the departments in the firm. The research team consulted with HR executives in each company to randomly select 3–10 employees from each participating department and invited them to complete the survey. All procedures performed in studies involving human participants were in accordance with the ethical standards of the institutional and/or national research committee and with the 1964 Helsinki Declaration and its later amendments or comparable ethical standards with written informed consent from all subjects. This research was approved by the Human Research Ethics Committee (HREC) at College of Economics and Management, Huazhong Agricultural University.

A total of 476 employees, 93 line managers, and 21 HR executives were invited to participate in the survey. We used a matched code to identify each employee’s response and that of the corresponding supervisor. We received responses from 427 employees, 84 line managers, and 21 HR executives, with a response rate of 89.71% for employees, 90.32% for line managers, and 100% for HR executives. After matching their responses, we obtained a final sample included 397 employees, 84 line managers, and 21 HR executives. Among 397 employees, about half of participants were male (52.90%); 81.61% had received college or undergraduate degrees, 5.54% had earned postgraduate degrees; their average age was 29.37 years old (*SD* = 5.91); their average tenure in the organization was 4.72 years (*SD* = 4.71). Among 21 firms, the average age of these firms was 16.53 years (*SD* = 17.13). The number of employees in most firms was less than 500 (61.90%).

### Measures

All of the original scales were developed in English and were presented in Chinese. To ensure the validity and reliability of scales, we used back-translation procedures.

#### High-Performance Work Systems

We adopted Jiang’s (2013) 18-item HPWS scale developed in the Chinese context (see Appendix). This scale measured HPWS using eight HR practices mentioned above: recruitment, selection, training, developmental performance management, performance-based compensation, flexible job design, participative decision-making, and information sharing. Organizational-level HPWS and employee experienced HPWS have different referent points. Hence, we modified the referents of organizational-level and employee experienced HPWS as “the employees of company” and “me,” respectively. HR executives and employees were requested to report organizational-level HPWS and employee experienced HPWS, respectively. A 5-point Likert scale ranging from 1 (strongly disagree) to 5 (strongly agree) was used. Following the prior literature (Liao et al., 2009; Messersmith et al., 2011; Aryee et al., 2012; Chang et al., 2014), we used an additive approach to operationalizing HPWS by calculating the mean scores of all HR practices. The Cronbach’s alpha for organizational-level HPWS and employee experienced HPWS scale were 0.87 and 0.94, respectively.

To ensure that the additive approach captured the concept better than other possible approaches (see Chadwick, 2010; Chang et al., 2014), we categorized the 18 items used to measure employee experienced HPWS into three bundles based on the AMO (ability-motivation-opportunity) framework (Appelbaum et al., 2000).^1^ Subsequently, we constructed interaction terms among the three bundles. Results showed that all of the two-way interactions or the three-way interaction was not significantly related to job performance and job satisfaction. These results indicated that adopting the additive approach to conceptualize HPWS was appropriate for the current study.^2^

#### Job Performance

We measured job performance using four items from Chen’s et al. (2002) scale. This scale was evaluated by line managers based on a 7-point response scale ranging from 1 (strongly disagree) to 7 (strongly agree). A sample item is “Always completes job assignments on time.” The coefficient alpha for the scale was 0.90.

#### Job Satisfaction

Job satisfaction was measured with a three-item scale from Erdogan and Bauer (2010). This scale was assessed by employees using a 5-point Likert scale ranging from 1 (strongly disagree) to 5 (strongly agree). A sample item is “All in all, I am satisfied with my job.” The coefficient alpha for the scale was 0.92.

#### Organization-Based Self-Esteem

Ten items adopted from Pierce et al. (1989) were used to measure OBSE. This scale was reported by employees using a 5-point response scale ranging from 1 (strongly disagree) to 5 (strongly agree). A sample item is “I am helpful around here.” The Cronbach’s alpha coefficient was 0.92.

#### Line Managers’ Goal Congruence

We used a three-item measure developed by Ozcelik (2013) to assess line managers’ goal congruence (1 = strongly disagree, 5 = strongly agree). A sample item is “My goals match or fit the goals of this organization and its current employees.” The Cronbach’s alpha coefficient was 0.86.

#### Control Variables

In addition to employee demographic variables such as gender, age, education level, and organizational tenure, we also controlled for firm age and firm size. Research has shown that larger firms may be more likely to use better developed HR practices and may facilitate employee performance (Sun et al., 2007). Firm age was included as a control variable because firm age was involved with evolution or adoption of HR practices and learning curve advantages in performance (Guthrie, 2001).^3^ Moreover, previous study has suggested that social exchange mediates the effects of HPWS (Takeuchi et al., 2007). Therefore, we controlled for social exchange. We measured this variable using an eight-item scale developed by Shore et al. (2006). This scale was rated by employees using a 5-point Likert scale (from 1 = strongly disagree to 5 = strongly agree). A sample item is “My organization has made a significant investment in me.” The Cronbach’s alpha for this scale was 0.76.

### Analytical Approach

The present data have a nested structure as employees are nested in groups, and groups are nested in firms. Thus, we adopted Hierarchical Linear Modeling 3 (HLM3) with HLM software to test our proposed hypotheses. To justify that HLM 3 is appropriate for analyzing three-level data, we ran null models with employee experienced HPWS, OBSE, job performance, and job satisfaction as the dependent variables, respectively. The results showed that the within-group, between-group, and between-firm variance of employee experienced HPWS were 0.32, 0.21, and 0.11, respectively. ICC(1)_firm_ was 0.17, indicating 17% of variance residing in between-firms in employee experienced HPWS. As such, ICC(1)_firm_ for OBSE, job performance, and job satisfaction were 8.33, 9.16, and 7.38%, respectively. The results corroborate that HLM 3 should be applied to examine the multilevel hypotheses.

Hypothesis 1 was to test cross-level interaction effect between organizational-level HPWS and line managers’ goal congruence in relation to employee experienced HPWS. According to Hofmann and Gavin (1998), we used the group means centering approach for line managers’ goal congruence and added group means of line managers’ goal congruence, and the interaction term between organizational-level HPWS and group mean of line managers’ goal congruence when assessing cross-level interaction effect.

Our model involves testing the serial indirect effects of the interaction between organizational-level HPWS and line managers’ goal congruence on employee job performance and job satisfaction sequentially through employee experienced HPWS and OBSE (Hypothesis 3a and 3b). As Preacher and Selig (2012) and Zhang et al. (2014) suggested, we utilized a parametric bootstrap procedure written in R software to estimate bias-corrected confidence intervals for these serial indirect effects based on 20,000 Monte Carlo re-samples.

## Results

### Confirmatory Factor Analyses

We conducted confirmatory factor analyses (CFAs) to test the discriminant validity of individual-level variables included in our study: employee experienced HPWS, OBSE, social exchange, job performance, as well as job satisfaction. According to Little et al. (2002), we adopted the first-order dimensions of employee experienced HPWS as the indicators of their respective latent variables to construct eight parcels. Furthermore, as recommended by Bagozzi and Edwards (1998) (see also Lam’s et al. (2015) empirical study), we formed five parcels of items as indicators for OBSE by averaging the items with the highest and lowest loading.

**Table 1** shows the results of CFAs. Results demonstrated that our hypothesized five-factor model fit data better [*χ^2^*_(340)_ = 1020.45, RMSEA = 0.07, TLI = 0.91, CFI = 0.92] than four-factor model 1 [*Δχ^2^*_(4)_ = 623.1, *p* < 0.001, RMSEA = 0.10, TLI = 0.82, CFI = 0.84], four-factor model 2 [*Δχ^2^*_(4)_ = 920.85, *p* < 0.001, RMSEA = 0.11, TLI = 0.78, CFI = 0.80], two-factor model [*Δχ^2^*_(9)_ = 2115.59, *p* < 0.001, RMSEA = 0.14, TLI = 0.63, CFI = 0.65], and one-factor model [*Δχ^2^*_(10)_ = 2807.75, *p* < 0.001, RMSEA = 0.16, TLI = 0.53, CFI = 0.57]. These results provided support for the distinctiveness of five variables.

**Table 1:** Comparison of factor structures.

Model	*χ^2^*	*df*	*χ^2^*/*df*	*Δχ2*(*Δdf*)	RMSEA	TLI	CFI
Five-factor model	1020.45	340	3.00		0.07	0.91	0.92
Four-factor model 1	1643.55	344	4.78	623.1^∗∗∗^(4)	0.10	0.82	0.84
Four-factor model 2	1941.30	344	5.64	920.85^∗∗∗^(4)	0.11	0.78	0.80
Two-factor model	3136.04.	349	8.99	2115.59^∗∗∗^(9)	0.14	0.63	0.65
One-factor model	3828.20	350	10.94	2807.75^∗∗∗^(10)	0.16	0.53	0.57

*N = 397. RMSEA, root mean square error of approximation. TLI, Tucker-Lewis index. CFI, comparative fit index. Five-factor model: employee experienced HPWS, OBSE, social exchange, job performance, and job satisfaction. Four-factor model 1: OBSE and social exchange were combined into one factor. Four-factor model 2: employee experienced HPWS and job performance were combined into one factor. Two-factor model: employee experienced HPWS, job performance and job satisfaction were combined into one factor; OBSE and social exchange were combined into one factor. One-factor model: all five factors were combined into one factor.*

### Descriptive Statistics

**Table 2** presents the means, standard deviations, and correlations for all study variables. Employee experienced HPWS were positively associated with OBSE (*r* = 0.48, *p* < 0.001), job performance (*r* = 0.29, *p* < 0.001), and job satisfaction (*r* = 0.61, *p* < 0.001). OBSE was positively related to job performance (*r* = 0.22, *p* < 0.001) and job satisfaction (*r* = 0.44, *p* < 0.001).

**Table 2:** Means, standard deviations, and correlations among study variables.

Variables	*M*	*SD*	1	2	3	4	5	6	7	8
**Level 1**
(1) Gender	0.47	—								
(2) Age	29.37	5.91	0.04							
(3) Education level	2.91	0.48	0.05	-0.09						
(4) Tenure	4.72	4.71	0.04	0.71^∗∗∗^	-0.13^∗^					
(5) Social exchange	3.58	0.72	-0.03	-0.07	0.10^∗^	-0.13^∗^				
(6) Employee experienced HPWS	3.77	0.77	-0.03	-0.06	-0.03	-0.18^∗∗∗^	0.62^∗∗∗^			
(7) OBSE	3.41	0.69	-0.03	-0.06	0.05	-0.05	0.54^∗∗∗^	0.48^∗∗∗^		
(8) Job performance	5.56	1.08	0.04	0.13^∗∗^	0.02	0.15^∗∗^	0.21^∗∗∗^	0.29^∗∗∗^	0.22^∗∗∗^	
(9) Job satisfaction	3.96	0.89	0.03	0.06	-0.04	-0.04	0.63^∗∗∗^	0.61^∗∗∗^	0.44^∗∗∗^	0.25^∗∗∗^
**Level 2**										
(1) Goal congruence	4.09	0.84								
**Level 3**										
(1) Firm size	1.43	0.60								
(2) Firm age	16.53	17.13	0.05							
(3) Organizational-level HPWS	4.25	0.47	-0.25	-0.18						

*N = 397 at Level 1, N = 84 at Level 2, N = 21 at Level 3. Gender: 0 = male, 1 = female. Education level: 1 = junior high school and below, 2 = senior high school, 3 = college or undergraduate, 4 = postgraduate and over. Firm size: 1 = less than 500, 2 = between 500 and 2000, 3 = more than 2000. ^∗^p < 0.05. ^∗∗^p < 0.01. ^∗∗∗^p < 0.001.*

### Hypotheses Testing

**Table 3** displays the results of the HLM analyses. Hypotheses 1 proposed the cross-level interaction effect of organizational-level HPWS and line managers’ goal congruence on employee experienced HPWS. In Model 9 we added gender, age, education level, and tenure at Level 1 and firm size and firm age at Level 3 as control variables and added organizational-level HPWS as a Level 3 predictor.^4^ The results of Model 9 in **Table 3** showed that the effect of organizational-level HPWS on employee experienced HPWS was marginally significant (Model 9, *γ* = 0.104, *p* < 0.1). Results for testing Hypothesis 1 are shown in Model 10, the cross-level interaction between organizational-level HPWS and line managers’ goal congruence was positively related to employee experienced HPWS (Model 10, *γ* = 0.11, *p* < 0.05), controlling for group means of line managers’ goal congruence, and the interaction term between organizational-level HPWS and group mean of line managers’ goal congruence as level 3 predictors.

**Table 3:** Results of HLM analyses.

Variables	Job performance			Job satisfaction			OBSE		Employee experienced HPWS	
	Model 1	Model 2	Model 3	Model 4	Model 5	Model 6	Model 7	Model 8	Model 9	Model 10
**Level 1**										
Intercept	5.60*** (0.09)	5.60*** (0.09)	5.59*** (0.06)	3.99*** (0.03)	3.98*** (0.04)	3.96*** (0.04)	3.39*** (0.03)	3.41*** (0.04)	3.76*** (0.07)	3.73*** (0.07)
Gender	-0.05 (0.08)	-0.04 (0.07)	-0.04 (0.07)	0.04 (0.04)	0.05 (0.05)	0.09* (0.04)	-0.074 (0.044)	-0.14* (0.05)	-0.05 (0.06)	-0.03 (0.07)
Age	0.001 (0.013)	0.001 (0.013)	-0.005 (0.013)	0.01 (0.01)	0.01 (0.01)	0.01 (0.01)	-0.018 (0.01)	-0.023* (0.01)	-0.01 (0.01)	-0.015 (0.016)
Education level	0.10 (0.16)	0.09 (0.16)	0.06 (0.15)	-0.02 (0.06)	-0.01 (0.06)	-0.064* (0.024)	0.11 (0.07)	0.07 (0.08)	-0.13 (0.10)	-0.12 (0.14)
Tenure	0.015 (0.013)	0.01 (0.01)	0.01 (0.01)	-0.003 (0.012)	-0.001 (0.01)	-0.002 (0.01)	0.01 (0.01)	0.019 (0.01)	-0.003 (0.013)	0.009 (0.01)
Social exchange		0.15 (0.10)	-0.09 (0.08)		0.53*** (0.07)	0.52***(0.08)				
Employee experienced HPWS	0.20* (0.09)	0.09 (0.09)	0.06 (0.09)	0.67*** (0.07)	0.32*** (0.08)	0.33***(0.07)	0.41*** (0.04)	0.39*** (0.07)		
OBSE		0.11* (0.05)	0.12* (0.05)		0.15* (0.06)	0.15* (0.06)				
**Level 2**										
Goal congruence			0.50*** (0.12)					0.123 (0.059)		0.25** (0.07)
**Level 3**										
Firm size	-0.08 (0.14)	-0.04 (0.14)	0.04 (0.10)	-0.001 (0.042)	-0.03 (0.04)	-0.01 (0.03)	-0.04 (0.04)	-0.09 (0.05)	-0.22* (0.09)	-0.175* (0.081)
Firm age	-0.001 (0.004)	0.0001 (0.004)	0.005 (0.004)	0.006 (0.003)	0.004 (0.003)	-0.001 (0.002)	-0.006** (0.002)	-0.008* (0.003)	-0.015*** (0.003)	-0.014** (0.004)
Organizational-level HPWS			0.19***(0.04)			0.10* (0.04)		0.03 (0.02)	0.104 (0.055)	0.082 (0.066)
Mean goal congruence			0.27* (0.10)			0.23*** (0.04)		0.121 (0.059)		-0.08 (0.10)
Organi zational-level HPWS × Mean goal congruence			0.39***(0.08)			-0.097 (0.048)		0.17* (0.06)		-0.02 (0.12)
**Cross-level interaction**										
Organizational-level HPWS × Goal congruence			0.19 (0.15)			0.01 (0.07)		0.08 (0.05)		0.11* (0.05)

*N = 397 at Level 1, N = 84 at Level 2, N = 21 at Level 3. Unstandardized coefficients were presented and the corresponding standard errors were reported in the parentheses. ^∗^p < 0.05. ^∗∗^p < 0.01. ^∗∗∗^p < 0.001.*

**FIGURE 2:**
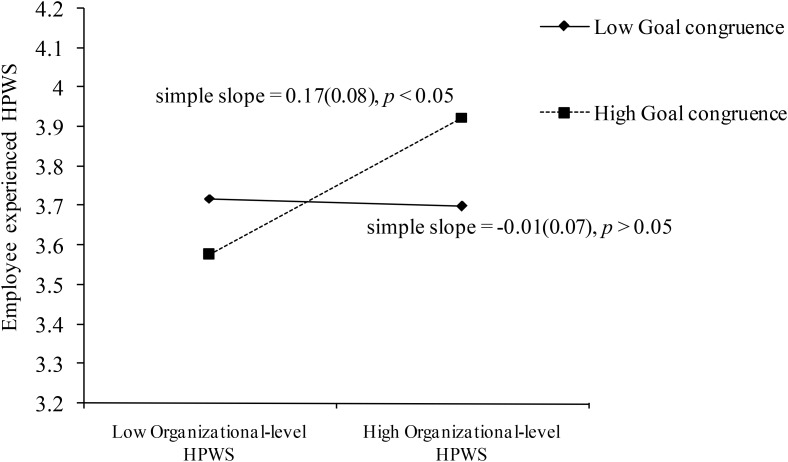
The interaction effect of organizational-level HPWS and line managers’ goal congruence on employee experienced HPWS. Values in parentheses are standard errors. *N* = 397 at Level 1, *N* = 84 at Level 2, *N* = 21 at Level 3.

To depict the moderating effect of line managers’ goal congruence, we plotted this moderating effect and calculated the simple slopes adopting Aiken and West’s (1991) procedure. **Figure 2** showed that there was a significant and positive relationship (*simple slope* = 0.17, *p* < 0.05) between organizational-level HPWS and employee experienced HPWS when line managers’ goal congruence was high (1 *SD* above mean), but a non-significant relationship (*simple slope* = -0.01, *ns*) when line managers’ goal congruence was low (1 *SD* below mean). Thus, Hypothesis 1 was supported.

Hypothesis 2a and 2b suggested that OBSE would mediate the effects of employee experienced HPWS on job performance and job satisfaction. We first ran Model 1 and Model 4 with job performance and job satisfaction as the dependent variables, respectively. We added control variables and employee experienced HPWS in these two models. **Table 3** showed that employee experienced HPWS positively predicted job performance (Model 1, *γ* = 0.20, *p* < 0.05) and job satisfaction (Model 4, *γ* = 0.67, *p* < 0.001). Model 7 indicated that employee experienced HPWS were positively related to OBSE (Model 7, *γ* = 0.41, *p* < 0.001). Next, in Model 2 and Model 5 we simultaneously added control variables, employee experienced HPWS, OBSE, and social exchange. Results demonstrated that OBSE was associated with higher job performance (Model 2, *γ* = 0.11, *p* < 0.05) and greater job satisfaction (Model 5, *γ* = 0.15, *p* < 0.05). Meanwhile, the effects of employee experienced HPWS on two individual outcomes (for job performance, *γ* = 0.09, *ns*; for job satisfaction, *γ* = 0.32, *p* < 0.001) became weaker than Model 1 and Model 4. Hence, Hypothesis 2a and 2b were supported.

In order to further test the significance of these indirect effects, we used a parametric bootstrap procedure recommended by Preacher and Selig (2012). Results revealed that the indirect effects of employee experienced HPWS on job performance (indirect effect = 0.045, 95% CI = [0.005, 0.087]) and job satisfaction (indirect effect = 0.062, 95% CI = [0.014, 0.113]) via OBSE were significant. Thus, Hypothesis 2a and 2b received further support.

Hypothesis 3a and 3b expected the serial indirect effects from the interaction between organizational-level HPWS and line managers’ goal congruence to employee experienced HPWS to OBSE and finally to job performance and job satisfaction. The examination of these serial indirect effects consisted of the product of the paths: (1) from the interaction term (organizational-level HPWS × line managers’ goal congruence) to employee experienced HPWS (Model 10, *γ* = 0.11, *p* < 0.05), (2) from employee experienced HPWS to OBSE (Model 8, *γ* = 0.39, *p* < 0.001), (3) from OBSE to job performance (Model 3, *γ* = 0.12, *p* < 0.05) and job satisfaction (Model 6, *γ* = 0.15, *p* < 0.05). We again adopted the parametric bootstrap procedure to test the significance of the serial indirect effects. Results showed that the indirect effects of the interaction between organizational-level HPWS and line managers’ goal congruence on job performance (indirect effect = 0.005, 95% CI = [0.0001, 0.013]) and job satisfaction (indirect effect = 0.006, 95% CI = [0.0002, 0.016]) through employee experienced HPWS and OBSE were significant and positive. Hence, Hypothesis 3a and 3b were supported.

## Discussion

Research has argued that a possible disconnection between organizational-level HPWS and employee experienced HPWS can occur (Nishii and Wright, 2008; Liao et al., 2009). However, few studies have examined how to align both. Our study extends the HPWS literature by testing how line managers’ goal congruence acts as a moderator in this relationship. Furthermore, we theorized and examined the mediating role of OBSE in the associations between employee experienced HPWS and job performance and job satisfaction (Pierce et al., 1989; Chan et al., 2013). Results demonstrated that line managers’ goal congruence strengthened the relationship between organizational-level HPWS and employee experienced HPWS, such that the relationship was significant and positive when line managers’ goal congruence was high, but a non-significant relationship when line managers’ goal congruence was low. In addition, employee experienced HPWS were related to job performance and job satisfaction, in part due to OBSE beyond social exchange. Ultimately, the interaction between organizational-level HPWS and line managers’ goal congruence indirectly affected employee job performance and job satisfaction sequentially through employee experienced HPWS and OBSE.

### Theoretical and Practical Implications

The study makes theoretical contributions in several ways. First, by examining the moderating role of line managers’ goal congruence, this research explores a boundary condition under which HPWS enacted by the organization are aligned with employee experienced HPWS, and addresses the inconclusive arguments and findings of this relationship. Prior research has found that a potential inconsistency between organizational-level HPWS and employee experienced HPWS may occur (Nishii and Wright, 2008; Liao et al., 2009). However, less study has investigated the factors that are implied within such a relationship. We hypothesized and indeed found that the influence of organizational-level HPWS on employee experienced HPWS depends on line managers’ goal congruence. Organizational-level HPWS and employee experienced HPWS became more aligned when line managers’ goal congruence was higher. Additionally, Liao et al. (2009) reported substantial variance in employee experienced HPWS at the within-group and between-group. Nishii and Wright (2008) argued that it is imperative to explore group-level factors that maximize the relationship between HR practices and performance. In the present research, we suggest that the impacts of organizational-level HR systems on individual outcomes are contingent on the variability in group-level factors such as line managers’ goal congruence, responding to the calls proposed in previous multilevel SHRM research.

Furthermore, our work also extends existing research that focused either on HPWS content or on the HPWS implementation process (Bowen and Ostroff, 2004; Liao et al., 2009; Aryee et al., 2012). In the current study, firm-level and employee perceived HPWS represent content and line managers’ goal congruence reflects implementation process. Line managers whose goals are consistent with those of their organizations will effectively implement HPWS content. Our findings suggest that it is critical to take both content and implementation process into account.

Moreover, our research sheds light on the mediating mechanism through which employee experienced HPWS facilitate job performance and job satisfaction. Scholars have suggested that future study should be from different approaches to better unlock the process by which HPWS foster employee desirable behaviors and attitudes (Jiang et al., 2013). Previous research has mainly been based on social exchange theory to explore the underlying mechanism of HPWS (Takeuchi et al., 2007; Messersmith et al., 2011; Kehoe and Wright, 2013). However, this mediating relationship fails to fully capture the role of OBSE. While existing work has tested how social exchange mediates the influences of HPWS (Takeuchi et al., 2007), our findings show that OBSE is a crucial mediator in this process as well.

In addition, this study also contributes to the OBSE literature. Prior research has indicated that OBSE is a result of contextual factors and individual characteristics, such as perceived organizational support, organizational justice, delegation, self-efficacy, and internal locus of control (McAllister and Bigley, 2002; Pierce and Gardner, 2004; Chen and Aryee, 2007; Ferris et al., 2009). To date, there has been limited knowledge about whether HPWS can result in OBSE. Our findings suggest that employee perceived HPWS foster OBSE. The impact of HPWS on OBSE that we uncovered, adds to the literature on the antecedents of OBSE.

Finally, this study has important management implications for organizations. We confirm that line managers’ goal congruence amplifies the positive relationship between organizational-level HPWS and employee experienced HPWS. Hence, organizations may adopt some measures to foster line managers’ goal congruence. For instance, companies should take full account of the interests of managers and employees when designing organizational strategies. Besides, Hoffman et al. (2011) suggested that transformational leaders may be instrumental in aligning employees’ interests effectively with those of the organizations. Thus, firms should carry out the training program of leadership skills to create a climate that embraces transformational leadership. Additionally, equal attention should be focused on OBSE that also contributes to employee job performance and job satisfaction. Organizations may enhance OBSE which ensures that employees’ needs to belong and to maintain positive self-worth are satisfied.

### Study Limitations and Future Research Directions

Our study has a number of limitations that should be explored in future research. First, we collected data from multiple sources (i.e., employees, line managers, and HR executives) which mitigated the potential impacts of common method variance on our findings. However, the data we obtained were cross-sectional in nature that limited our ability to make conclusions about causal inferences. Thus, future research needs to adopt longitudinal research designs to rigorously test the hypothesized relationships over time.

Second, as the study was conducted in China, the generalizability of our findings to other cultural contexts remains an empirical question. Therefore, future research should examine whether our findings are also applicable to other parts of the world.

Third, we treated HPWS as an overall configuration or aggregation of HR practices in the current research. Such approach that has been used in prior SHRM study did pose weakness to the current research. For example, employees may be more sensitive to certain HR practices. Therefore, they are more likely to inquire the information about these HR practices and have similar perceptions to their organizations. Future research should test how the characteristics of HR practices and individual factors influence the relationship between organizational-level HPWS and employee experienced HPWS.

A fourth potential limitation of this study is that we rely on theoretical research and empirical evidence to guide the prediction that line managers’ goal congruence moderates the relationship between organizational-level HPWS and employee experienced HPWS through facilitating line managers’ implementation of HPWS. We, however, did not directly measure line managers’ implementation efforts. Future research should extend this study and examine whether line managers’ goal congruence promotes their implementation efforts, and which further strengthens the effect of organizational-level HPWS on employee experienced HPWS. Moreover, we only focused on line managers’ goal congruence, there may be other moderators that should be examined in future study, such as organizational slack resources, the communication strategies and commitment in transferring HPWS to employees from the management.

Finally, our study only explored the mediating role of OBSE in the relationships between employee experienced HPWS and individual outcomes, other approaches may also be used to explain such associations, such as basic psychological needs. According to self-determination theory, individuals have three basic psychological needs that include competence, autonomy, and relatedness. The satisfaction of these three basic psychological needs may promote employees’ positive behaviors and attitudes (Ryan and Deci, 2000). HPWS can improve employees’ knowledge, skills, and abilities, stimulate their task motivations, and make autonomy available for employees at work, hence satisfy their basic psychological needs. Consequently, future research should explore how basic psychological needs mediate the effects of HPWS.

## Conclusion

In closing, the primary goal of this study was to examine how and when organizational-level HPWS affect employee job performance and job satisfaction. With multilevel multisource data, the results showed that line managers’ goal congruence reduces the gap between organizational-level HPWS and employee experienced HPWS. We also found that employee experienced HPWS drive job performance and job satisfaction through the mechanism of OBSE beyond social exchange. Our study not only advanced new knowledge concerning how HPWS influence employee outcomes but also inspired scholars to explore additional boundary conditions and explanatory mechanisms in relation to the HPWS-individual outcomes associations.

## Author Contributions

JZ: conceived and designed the theoretical model and wrote the first manuscript. MA: analyzed the data. PB: conceived and designed the theoretical model. YZ: performed the survey. UT: improved the manuscript.

## Conflict of Interest Statement

The authors declare that the research was conducted in the absence of any commercial or financial relationships that could be construed as a potential conflict of interest.

## Footnotes

1 Scholars have drawn upon the AMO (ability-motivation-opportunity) framework of HRM to decompose HPWS into three components: ability-enhancing HR practices, motivation-enhancing HR practices, and opportunity-enhancing HR practices. Ability-enhancing HR practices include recruitment, selection, and training. Developmental performance management and performance-based compensation act as motivation-enhancing HR practices. Flexible job design, participative decision-making, and information sharing are important components of opportunity-enhancing HR practices (Jiang et al., 2012).

2 We thank the reviewer for this suggestion.

3 Learning curve shows a non-linear relationship in which the performance enhances with practices (Trappey et al., 2016). In addition to task proficiency, the improvement of management techniques, extensive training, reward, and benefits are also key determinants of learning curve.

4 We thank the reviewer for this suggestion. **Table 2** reported that the correlation coefficient between age and tenure was 0.71, which might cause collinearity issues and make part of the estimation statistically inaccurate. We found essentially the same patterns of results for our hypothesized relationships when controlling for age or tenure, or both.
