# Supplementary material for: How Do High-Performance Work Systems Affect Individual Outcomes: A Multilevel Perspective
Source: Front Psychol. 2018 Apr 24;9:586. doi: 10.3389/fpsyg.2018.00586 (PMC5930235; doi:10.3389/fpsyg.2018.00586)
Supplement: Supplementary file 1 [file Data_Sheet_1.docx]

**APPENDIX**

**Organizational-Level High-Performance Work Systems**

The following items refer to how employees are managed in your company. Please indicate the extent of your agreement or disagreement about each statement.

1. Selection emphasizes traits and abilities required for providing high quality of performance.

2. Recruitment process uses many different recruiting sources (agencies, universities, etc.).

3. Selection process is comprehensive (uses interviews, tests, etc.).

4. The company provides an orientation program for newcomers to learn about the company.

5. The company continuously provides training programs.

6. The company invests considerable time and money in training.

7. Performance appraisals provide employees feedback for personal development.

8. Performance appraisals are based on objective, quantifiable results.

9. Supervisors get together with employees to set their personal goals.

10. Employee salaries and rewards are determined by their performance.

11. The company attaches importance to the fairness of compensation/rewards.

12. Employees receive monetary or nonmonetary rewards for great effort and good performance.

13. The company considers employee off-work situations (family, school, etc.) when making schedules.

14. The company has its ways or methods to help employees alleviate work stress.

15. The company has formal grievance procedures to take care of employee complaints or appeals.

16. If a decision made might affect employees, the company asks them for opinions in advance.

17. Employees are often asked to participate in work-related decisions.

18. The company shares job-related information with employees (e.g., company operation, sales, etc.).

**Employee Experienced High-Performance Work Systems**

The following items refer to HR practices that your company implements for you. Please indicate the extent of your agreement or disagreement about each statement.

1. Selection emphasizes traits and abilities required for providing high quality of performance.

2. Recruitment process uses many different recruiting sources (agencies, universities, etc.).

3. Selection process is comprehensive (uses interviews, tests, etc.).

4. The company provides an orientation program for me to learn about the company.

5. The company continuously provides training programs for me.

6. The company invests considerable time and money in training for me.

7. Performance appraisals provide feedback for my personal development.

8. Performance appraisals are based on objective, quantifiable results.

9. Supervisors get together with me to set my personal goals.

10. My salaries and rewards are determined by my performance.

11. The company attaches importance to the fairness of compensation/rewards.

12. I receive monetary or nonmonetary rewards for great effort and good performance.

13. The company considers my off-work situations (family, school, etc.) when making schedules.

14. The company has its ways or methods to help me alleviate work stress.

15. The company has formal grievance procedures to take care of my complaints or appeals.

16. If a decision made might affect me, the company asks me for opinions in advance.

17. I am often asked to participate in work-related decisions.

18. The company shares job-related information with me (e.g
